# Technical-Tactical Behaviors Analysis of Male and Female Judo Cadets’ Combats

**DOI:** 10.3389/fpsyg.2020.01389

**Published:** 2020-06-19

**Authors:** Bianca Miarka, Diego Ignácio Valenzuela Pérez, Esteban Aedo-Muñoz, Lucas Oliveira Fernandes da Costa, Ciro José Brito

**Affiliations:** ^1^Laboratory of Psychophysiology and Performance in Sports & Combats, School of Physical Education and Sport, Federal University of Rio de Janeiro, Rio de Janeiro, Brazil; ^2^Laboratory of Physiological and Motor Analysis in Health and Performance of the Physical Education Department, Federal University of Juiz de Fora, Governador Valadares, Brazil; ^3^Escuela de Kinesiologia, Facultad de Salud, Universidad Santo Tomás, Santiago, Chile; ^4^Biomechanics Laboratory, Chilean High-Performance Center, Physical Activity, Sport and Health Sciences Laboratory, Universidad de Santiago de Chile, Santiago, Chile; ^5^Physical Education Department, Universidad Metropolitana de Ciencias de la Educación, Santiago, Chile

**Keywords:** motor control, sport psychology, task performance and analysis, martial arts, gender

## Abstract

This brief research report showed technical-tactical behaviors of male and female judo cadets during combats, comparing the frequency and time of judo combat actions, techniques and penalties. The data was composed for 3,240 sequential technical-tactical behavior analysis from 108 female and 300 male cadet combats recorded of public judo championships. Combat, standing combat moments, approach action, gripping action, attack, groundwork actions and pause moment were observed and determinant technical-tactical behaviors (frequencies of actions, penalties and type of attacks) analysis were done with FRAMI software, followed by Mann-Whitney and Student’s *t*-test, *p* ≤ 0.05. Our main results indicated that male cadets with 58.66s ± 50.26s demonstrated longer gripping action than female with 38.44s ± 30.44s, as standing combat (*tachi-waza*) had differences between male with 96.8s ± 72s and female athletes with 75.85s ± 56.97s. Moreover, male cadets had higher sacrifice techniques (*sutemi-waza*) actions than female athletes. This information could be used to a best performance associated with “psyching-up” as much as it could be used on physical training and technical-tactical ability of female and male cadets.

## Introduction

Gender has been recognized as a major difference of combat behavior over the effect of anthropometric and physical condition features related to the genetic and hormonal variances (Koyama et al., 2019; Schor et al., 2019). Some authors have suggested that gender differences in behavior conditions during combats may increase in judo (Suzuki et al., 2019); other have studied physiological sex differences in combat sports, but with limited behavior data. However, men and women differences should first be studied through the complete panel of quantifiable behavior sequential analysis. Numerous coaches around the world have experienced that best performance associated with psyching-up strategies on the physical and technical-tactical training (Tod et al., 2015). Cognitive approaches are consistently associated with behavior analysis and improved performance (results range from 61 to 65%) (Tod et al., 2015). However, there is a lack of information for the practical application of sport psychology methods to acquire and retain new skills in cadet’s judo, as well as to modify behaviors which reduce performance.

For instance, technical-tactical analysis in judo emphasis on recognizing sequential behavior arrangements, often denoted to as “indicators,” in the competitive situation (Brito et al., 2017). The performance in judo is associated with the ability of a male or female athlete to execute specific actions at the correct moment during each combat moment while quickly adapting to the constantly fluctuating combat environment to the next action (Brito et al., 2017). Technical-tactical behavior analysis have been well-described in judo, verifying match demands (Coswig et al., 2018), techniques and muscle group specific torque production (Lech et al., 2015). Specifically, during female and male judo adult’s competition, technical-tactical actions of different technical-tactical behaviors and pause strategies are highly varied and the associations between these variables together describe a judo’s system of attack or fighting style (López Díaz-de-Durana et al., 2018). Despite this important finding about the senior age class, until now, it is not very clear whether there are technical-tactical behavior differences between female and male youngers.

Particular behavior patterns that could determine different male and female cadet performance, considering maturational state and age effects (Fukuda, 2015), such as in senior, the approach trying to grip, gripping behaviors (*kumi-kata*), and scored attacks with different combinations (Coswig et al., 2018). Pinelopi Stavrinou et al. (2017) indicated a high influence that several judo bouts have in perceived exertion and cardiovascular system, highlighting the anaerobic pathway as the main metabolic involvement during four simulated judo combats, with about 11 attacks on standing combat (*tachi-waza*) and one attack on groundwork combat (*ne-waza*) per combat. Preceding authors demonstrated the use of specific effective tactical behaviors in high level cadet judo athletes, as re-gripping patterns during the gripping action (Ito et al., 2019). Despite this important finding, little is known about the differences between men and women cadets in relation to the behaviors of the gripping action. Female judo cadets training has largely been ignored throughout the youth training programs and studies about behavior judo analysis. Cadets judo combat research, programming and interventions have focused almost exclusively on men athletes with little attempt to understand the gender specific needs of women younger practitioners. Therefore, individual training programs could be adjusted for male and female judo athletes with consideration for the technical-tactical behavior and temporal structure of the competitive environment (Sterkowicz-Przybycien et al., 2017).

Regarding senior class, attacks from the same gripping preparation increase probabilities of resulting in a successful score for both male and female athletes (Muddle et al., 2017). The gripping moment seem to be associated with sequential actions during judo combat and critical indicators (Calmet et al., 2006). Other research indicated that percentage of total time spent by beginners with 20 h of practice and judo black-belt experts gripping the opponent’s judogi with both hands, attacking and throwing were ∼90 and ∼25% respectively, indicating differences associated with accumulated time of practice (Calmet et al., 2010). This phenomenon has not been investigated in cadet’s judo championships, considering female and male combats. A full literature review did not produce any research addressing the ability to integrate gripping time and judo behaviors based on juvenile actions during competitions. Comparing genders.

In the same way, the gripping demand that occurs in the senior class shows specifics tactical arrangements with stances opposite from their opponents (*kenka-yotsu*) to preserve a defensive situation preceding to attack or counter-attack (Ito et al., 2019). A recent research suggested that motor action analysis in judo be conducted with consideration for sex modifications in senior combat actions with a wide range of interconnected behavior components (Sterkowicz-Przybycien et al., 2017), such as the approach action (Sterkowicz-Przybycien et al., 2017), attack with the type of technique (Anthierens et al., 2019), groundwork attempts (Brito et al., 2017) and pause phase analysis (Sterkowicz-Przybycien et al., 2017). Currently, the specific behaviors of each gender in cadet class remain unknown; this knowledge is essential to target technical-tactical training, considering each combat phase. Therefore, the purpose of the present research was to report technical-tactical behaviors, comparing occurrences and respective time of judo combat actions, scores and penalties used by female and male cadet athletes during championships.

## Methods

### Sample

The data was composed for 3,240 sequential technical-tactical behavior analysis from 108 female and 300 male cadet combats recorded of public judo championships. In order to be included, each video had to be with a minimal standard definition of 480/60i and taken from a landscape assessment of the entire competition area. The competitive bouts were evaluated following previously outlined protocols (López Díaz-de-Durana et al., 2018), from four local competitions. The VirtualDub Program 1.8.6(2) was used to fragment and edit records and the validated judo match analysis, Frami^®^ software (Miarka et al., 2011), was utilized to make the technical-tactical behavior analysis; the present documentary research guaranteed confidentiality and anonymity by replacing the athletes’ personal ID. All participants had previous experience (>2 years) with official judo events (range level: local to national), rules and procedures (International Judo Federation (IJF), 2018). No modifications were made in the judo training (range frequency: two to five times/week, range time: three to 12 h/week) nutritional or hydration status of participants, following preceding protocols (Antonietto et al., 2019). There are no ethical problems in investigating data of public events, as disposed by prior protocols (Sterkowicz-Przybycien et al., 2017). Present research was earlier approved by the local Ethics and Research Committee, following WMA Declaration of Helsinki.

### Technical-Tactical Behaviors Analysis and Reliability Testing

**FIGURE 1:**
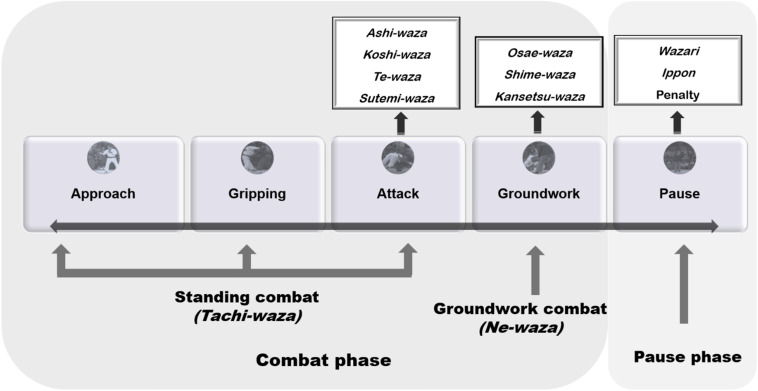
Behavior judo combat model analysis.

A behavior analysis of combat states was performed according to each frequency of occurrence and time, then normalized and presented by the combat and pause ratio. The analysis was defined in periods of the combat actions: approach, gripping, attack, groundwork and pause, following preceding reports (Sterkowicz-Przybycien et al., 2017). So, the approach was well-defined as the moment between the indication from the referee announcing the start of contest (*hajime*) and the realization of the grip (*kumi-kata*). During this moment, participants do not have any contact between them (Calmet et al., 2010). Gripping action was demarcated as the moment between the accomplishment of the grip (*kumi-kata*) and the breaking of interaction with the adversary *judogi* (Miarka et al., 2011). Attack was demarcated as the preliminary breaking balance of the adversary (*kuzushi*) and the preparatory changes of the body, particularly of the feet, completed prior to the attack (*tsukuri*) and the final displacement, trying to throw the opponent (*kake*) (Miarka et al., 2015). The groundwork combat was the moment where one or both fighters executed groundwork actions (*ne-waza*), according to previous protocols (Sterkowicz-Przybycien et al., 2017). The pause was the moment between the signal for contest pause (“*matte*”) and the signal to restart the contest, with a voice command (*hajime*). The present study did not verify the “*sono-mama*” and “*yoshi*” commands halting and restarting the combat, which would also have been analyzed as recovery if they had had happened (Sterkowicz-Przybycien et al., 2017). Lastly, combat action or phase was the moment between the order from the order to initiate the combat (*hajime*), the order to stop the combat (*matte*), and the order to finish the combat (*soremade*), according to previous protocols (Sterkowicz-Przybycien et al., 2017). Figure 1 demonstrates the behavior analysis model. Attacks that have been prohibited by rules have not been documented.

The index and classification of Kappa values of technical-tactical behavior analysis used in the present study for Inter-expert and Intra-expert measurements were 0.74 and 0.82, classified as “Strong” and “Almost perfect” for Approach Action, 0.45 and 0.96, classified as “Moderate” and “Almost perfect” for Gripping Action, 0.52 and 0.97, classified as “Moderate” and “Almost perfect” for Attack Action, 0.84 and 0.90, classified as “Almost perfect” and “Almost perfect” for Groundwork Actions and 0.91 and 0.99, classified as “Almost perfect” and “Almost perfect” for Pause Phase. The classification of *nage-waza* (throwing techniques) and *katame-waza* (groundwork techniques) attacks considered the *nage-waza* techniques classification of Kodokan, correspondingly in the procedure of the four throwing types: *te-waza* (arm techniques) with 0.75 and 1.00 classified as substantial, *ashi-waza* (leg/foot technique) with 0.97 and 1.00 classified as “Almost Perfect,” *koshi-waza* (hip technique) with 1.0 and 1.00 classified as “Almost Perfect,” and *sutemi-waza* (sacrifice technique) with 0.96 and 0.95 classified as “Almost Perfect,” Mean-Whitney comparisons did not show effects between intra or inter measurements, *p* ≤ 0.05, following preceding indications (Dal Bello et al., 2019).

### Statistical Analysis

The SPSS 20.0 for Windows was used to obtain descriptive and statistical inferences. Descriptive statistics were demonstrated as mean and standard deviation (SD), and Independent T-tests conducted to compare time-motion variables. While frequencies were showed as median, mean [25th percentile; 75th percentile] values and Mann-Whitney tests were used to compare combat phase and technical-tactical frequencies between male and female judo cadets. Effect size was verified following preceding reports (Brito et al., 2017), considering the significance level of *p* ≤ 0.05.

## Results

Comparisons of time-motion analysis of judo actions between male and female cadets are in Table 1.

**TABLE 1:** Comparisons of behavior judo combat analysis of female versus male cadets (time).

Technical-tactical actions	Group	Mean ± SD	*t*	*df*	*p*-value	95% CI
						Lower; Upper
Combat moment(s)	Female	163.5 ± 135.9	−1.168	402	0.24	−48.0; 12.2
	Male	181.4 ± 134.2	−1.161	177.408		−48.3; 12.5
Standing combat(s) (*Tachi-waza*)	Female	**75.9** ± **57.0**	**−2.688**	**402**	**0.007**	**−36.3; −5.6**
	Male	96.8 ± 72.0	−3.008	225.014		−34.7; −7.2
Approach action(s)	Female	31.3 ± 26.5	−0.085	402	0.93	−6.3; 5.7
	Male	31.5 ± 26.9	−0.085	181.501		−6.2; 5.7
Gripping action(s)	Female	**38.4** ± **30.4**	**−3.862**	**402**	**≥0.001**	**−30.5**; **−9.9**
	Male	58.7 ± 50.3	−4.857	298.047		−28.4; −12.0
Attack action(s)	Female	4.3 ± 4.8	−1.380	402	0.16	−1.9; 0.3
	Male	5.1 ± 5.0	−1.396	183.027		−1.9; 0.3
Groundwork actions(s) *Ne-waza*	Female	40.8 ± 35.2	0.825	402	0.41	−4.4; 10.7
	Male	37.7 ± 33.0	0.799	169.778		−4.6; 10.9
Pause moment(s)	Female	46.8 ± 76.7	0.098	402	0.92	−12.4; 13.7
	Male	46.1 ± 50.6	0.080	135.290		−15.3; 16.6

*Variables with significant difference are in bold.*

Male cadet judo athletes presented longer gripping action than female fighters and, longer *tachi-waza* moment.

Table 2 shows technical-tactical behaviors of cadets during judo championships.

**TABLE 2:** Comparisons of technical-tactical behaviors judo combat of female versus male cadets (frequencies).

Technical-tactical actions	Female	Male	*U*	*Z*	*p*-value	ES
	M(Q1;Q3)	M(Q1;Q3)				
**Combat phase**	4 (2;10)	5 (3;10)	145690	−1.008	0.31	0.05
***Tachi-waza* phase**	4 (2;10)	5 (3;10)	145690	−1.008	0.31	0.05
*Ashi-waza*	1 (0.02;3)	1 (0.03;3)	15614	−0.574	0.56	−0.03
*Koshi-waza*	0.05 (0;1)	0.05 (0;1)	16028.5	−0.204	0.83	−0.01
*Te-waza*	0.05 (0;1)	0.05 (0;1)	14993.5	−1.199	0.23	−0.06
***Sutemi-waza***	**0.02 (0;1)**	**0.04 (0;1)**	**14586**	**−1.824**	**0.06**	**−0.09**
***Ne-waza* phase**	3 (1;7)	4 (2;6)	148540	−0.731	0.46	0.04
*Osae-waza*	0 (0;0.5)	0 (0;0.5)	15660	−0.904	0.36	−0.04
*Shime-waza*	0 (0;0.1)	0 (0;0.3)	16014	−0.631	0.52	−0.03
*Kansetsu-waza*	0 (0;0)	0 (0;0)	16068	−0.469	0.63	−0.02
Pause phase	3 (1;9.5)	5 (2;8)	144380	−1.137	0.25	0.06
*Wazari* score	0.2 (0;0.75)	0.2 (0;0.7)	14946	−1.259	0.20	−0.06
*Ippon* score	0.3 (0;1)	0.3 (0;1)	15056	−0.659	0.51	−0.03
Penalty	0.3 (0;1)	0.4 (0;1)	14876	−1.412	0.15	−0.07

*Variables with significant difference are in bold.*

There was no significant difference for any comparison (*p* > 0.05).

## Discussion

Despite the amount of technical-tactical behaviors studies in judo, few evaluated cadet athletes. Technical-tactical behavior studies in this age group can help coaches to properly train and prepare their athletes for competitions. Therefore, the present study compared technical-tactical behavior analysis between male and female cadet judo combats. It was the first time, for the best of our knowledge, that the combination of technical-tactical actions that was presented comparing men and women cadets. The main results demonstrated a main difference between groups, male cadets with longer gripping action, *tachi-waza* moment and higher *sutemi-waza* frequencies than female athletes. This data can be used to create cognitive strategies on contextual and physical training (Tod et al., 2015). Mental strategies are consistently connected with ∼65% of improved performance (Tod et al., 2015). Present judo behavior model and results had emerged to bridge the gap between research and mental strategy practices for competitive events and rehabilitation, as technical and tactical behavior models associated with youngers were not available in scientific studies.

Regarding present results, the longer gripping moment and the use of both collar grip was recurrent in defensive situations, as it could maintain a high space control over the opponent, but it reduced opponent’s imbalance possibilities (*kuzushi*) and, consequently affected the execution of different techniques and biomechanical actions during collect data analysis. Cadet judo athletes demonstrated 50–60% of shorter *tachi-waza* moment than pre-juvenile, juvenile, junior and senior judokas at the same level (Miarka et al., 2012). In contrast, groundwork actions and pause moment analysis presented similar results when compared with male and female pre-juvenile, juvenile, junior and female senior judo athletes i.e., 42 ± 33s and 41 ± 31s, 37 ± 26s and 35 ± 26s, and 33 ± 26s and 41 ± 35s and 36 ± 31s (Miarka et al., 2014) – all them shorter than male senior athletes with 50 ± 37s (Miarka et al., 2012). This could be clarified by considering the modifications in the rules that were changed in order to make judo a more attractive sport to the public, consequently lower groundwork behaviors were observed in specific weight divisions (Sterkowicz-Przybycien et al., 2017). Furthermore, modifications in rules over the last years have proposed to enhance the dynamic of the combat, which may have impacted behavioral strategies, injuries frequencies and pause moments (Stephenson and Rossheim, 2018).

Preceding authors indicated that sport specialization of judo cadets was not related with success across an athletic career, finding indicated that less than 7% of the athletes repeated their initial state-level competitive outcomes after 10 years of judo competition (Julio et al., 2011). indeed, the evidence is contrary to this hypothesis as the pre-juvenile male age class had a lower percentage of medal winners in the last year of the 10 years follow-up, and male and female pre-juvenile age classes had a lower percentage of medal winners in the last 3 years of this follow-up than the grand mean (Julio et al., 2011). Therefore, the early specialization procedure in judo is not likely to be related with success in senior age class, and programs directed to talent promotion should emphasis the development of motor skills, contextual training and mental development than the competitive outcome (Julio et al., 2011). Concerning this programs, present research contributes with essential information for the development of motor skills, as well as the mental and physical training of youth practitioners, according to their match references, avoiding the use of senior match situations.

Our evaluated athletes showed a higher tendency to take with the traditional grip, right handgrip on the collar and left handgrip on the sleeve (*migi-kumi-katá*) and present a higher volume of *ashi-waza –* this type of technique is used twice as often when compared to other types during local/regional cadet judo championship. Previous studies reported a predominance of *te-waza* during high level senior athletes in the London Olympic Games, but with specific differences in the techniques applied (Sterkowicz-Przybycien et al., 2017). While authors observed higher use of *ashi-waza* in junior versus senior of state and national level female athletes (Kamitani et al., 2017). Male cadets presented higher frequencies of sacrifice techniques than female. The use of *sutemi-waza* has been described to happen more often in senior compared to junior athletes (Miarka et al., 2014) and may be chosen during male cadets’ championship due to greater scoring efficiency. *Ashi-waza* techniques could not result in higher scores, but it is known the higher attack volume can result in competitive advantage since will result in a penalty to the opponent (Brito et al., 2017), a factor that should be considered by coaches when designing the training and preparing competitive strategies of cadets.

Groundwork combat did not demonstrate higher predominance in recreational cadets’ level, while for high level female athletes, *osae-waza* was determinant as winning factor (Miarka et al., 2017). In fact, groundwork motor actions were linked to effective actions of 20% of all attempts to attack during the Olympic Games (Sterkowicz-Przybycien et al., 2017). In a previous study, senior judo matches presented a greater use of *osae-waza*, *shime-waza* and *kansetsu-waza* (Sterkowicz-Przybycien et al., 2017). In senior high-level athletes, the *osae-waza* increased the performance probability when combined with judo techniques that implicate rotation, for instance *morote-seoi-nage*, and sacrifice throws (*sutemi-waza*), for example *tomoe-nage* (Miarka et al., 2017). Previous findings indicated that the use of *ahi-waza* with Arm/foot lever to realize a fast transition to the groundwork could be responsible of ∼60% of the winning cases affecting positively the *osae-waza* (Miarka et al., 2017).

A potential limitation of behavior analysis is the consistency of the data achievement procedure, or the researcher’s capability to reproduce the detected behavior once measurement is repeated (Coswig et al., 2018). Large variations in the combat moment, frequency and mean duration of actions measured during reliability analyses can affect inter-observer consistency (Coswig et al., 2018). The observational-descriptive approach implemented in the current investigation may limit extrapolation of the results. Furthermore, cadet athletes likely produce dissimilar physical and physiological effects for a particular combat behavior than senior judo athletes (Coswig et al., 2018). During the present behavior analysis, weight divisions of female and male cadets demonstrated great temporal similarities, except for female half-heavyweight and heavyweight categories, in which *tachi-waza* (i.e., approach/gripping phases) was longer than half-lightweight division; however, without significant effects in the behavior frequencies, score or penalties for male and female younger judo athletes. This also indicates the need for future further research to verify how changes in regulation affect the cadet combat and their respective weight divisions and technical-tactical actions.

## Conclusion

Our results demonstrated the differences between men and women judo cadet athletes – with longer gripping action and *tachi-waza* preference of male than female combats. These differences among groups need to be reflected in judo physical and mental training practices. Our findings emphasize the importance of accounting the frequency and time of technical-tactical behaviors and the singularities of each gender. Therefore, psychologists and coaches can make extensive use of behavior analysis during championships and data collection measures to deliver advantageous feedback about each combat action while developing systematic methods of mental and physical technical-tactical training, focusing on system of attack variations, using groundwork attacks (*katame-waza*), avoiding longer time with the same gripping behavior, which negatively influence the control over the opponent and it could increase the chance to receive penalties during cadets championships.

## Data Availability Statement

The raw data supporting the conclusions of this article will be made available by the authors, without undue reservation, to any qualified researcher.

## Ethics Statement

Present research was earlier approved by the local Ethics and Research Committee, following WMA Declaration of Helsinki. Written informed consent to participate in this study was provided by the patient/participants’ or patient/participants’ legal guardian/next of kin where required.

## Author Contributions

All authors listed have made a substantial, direct and intellectual contribution to the work, and approved it for publication.

## Conflict of Interest

The authors declare that the research was conducted in the absence of any commercial or financial relationships that could be construed as a potential conflict of interest.
